# Hyperspectral imaging for intraoperative brain tumor identification through fusion of spectral, textural, and spectral index features

**DOI:** 10.1371/journal.pone.0340879

**Published:** 2026-02-23

**Authors:** Jianhua Liu, Chenglong Zhang, Jinzhuang Xu, Luwei Wang, Mingzhong Pan, Xiaopeng Ma, Wenjun Xia

**Affiliations:** 1 Hangzhou Institute for Advanced Study, University of Chinese Academy of Sciences, Hangzhou, China; 2 School of Control Science and Engineering, Shandong University, Jinan, China; 3 Department of Intensive Care Unit, Shandong Provincial Hospital Affiliated to Shandong First Medical University, Jinan, Shandong, China; Michigan State University, UNITED STATES OF AMERICA

## Abstract

Brain tumor is a common neurological surgical disease, where surgical resection is the primary treatment method. Neurosurgeons need to accurately determine the location of the tumor during tumor resection surgery, but existing clinical tumor identification technologies face numerous challenges, such as high equipment costs, long processing times, a certain degree of invasiveness, and insufficient image clarity. In this work, we propose a hyperspectral image detection algorithm based on the fusion of multiple features to maximize the determination of tumor boundaries. The algorithm establishes the machine learning models of Support Vector Machine (SVM) and Random Forest (RF) by integrating data features from optimal wavelengths, spectral indices, and textural features. Experimental results show that on different datasets, the classification accuracy of the three-feature fusion model is significantly higher than that of models using only two features or a single feature. Hyperspectral tumor image recognition can effectively help distinguish the tumors from the surrounding tissue, thereby enhancing the safety and thoroughness of tumor surgery.

## Introduction

Surgical resection is the conventional treatment for brain tumors, but it requires doctors to identify and remove tumor tissue as accurately as possible from normal brain tissue, otherwise it not only increases the risk of complications such as intracranial bleeding and infection but can also lead to tumor recurrence [1–3]. Conversely, excessive resection may alter the distribution of intracranial pressure, causing symptoms like headaches, nausea, vomiting, and even lead to severe consequences such as paralysis, loss of sensation, and speech disorders.Therefore, precise identification of tumor location during surgery is crucial for assisting doctors in resection, which can minimize damage to normal brain tissue [4]. Currently, the main technologies used in clinics to identify tumor locations are surgical navigation [5], intraoperative ultrasound [6,7], and intraoperative biopsy [8]. But they have their respective limitations. Surgical navigation systems are costly and rely on preoperative images, which may not adapt in real-time to anatomical changes that occur during surgery; intraoperative ultrasound provides non-standard images that differ from the standard images familiar to neurosurgeons; intraoperative biopsies can cause damage to the patient, are time-consuming, and carry the risk of false negatives.

Given the shortcomings of existing technologies, a cost-effective, accurate, and non-invasive technology is needed to develop for tumor resection [9]. Hyperspectral Imaging (HSI), as an emerging imaging technology, can generate medical images with one-dimensional spectral information and two-dimensional spatial information [10] by capturing the reflective or transmissive characteristics of tissue across multiple spectral bands. Benefiting from the advantage of broad spectral range and high resolution, HSI can provide detailed information for identifying tumor locations at the molecular and cellular levels. In recent years, the application of hyperspectral technology in cancer detection has become increasingly widespread [11]. For example, Huang Yi proposed a supervised least squares support vector machine segmentation method based on feature spectra for the segmentation of malignant melanocytes, achieving a segmentation accuracy of 85% in experimental samples of cutaneous melanoma. Leon and colleagues developed a dermatological HSI acquisition system and proposed a semi-supervised algorithm-based processing framework for the automatic identification and classification of PSL, achieving the diagnosed results with 87.5% of sensitivity and 100% of specificity. These studies indicate that the combination of hyperspectral imaging and machine learning technology is expected to provide an accurate technology for determining the boundaries of intraoperative brain tumors [12,13].

In this study, we proposed a hyperspectral imaging detection framework for brain tumor based on the fusion of multiple features to achieve precise judgment of tumor location and assist doctors in surgical resection. We first processed the data in three stages: using the DGWCR algorithm to select the optimal wavelengths (OWS), utilizing the Relief algorithm to choose appropriate spectral indices (SIS), and collecting textural features (TFS) through the IAPS algorithm. Then, we introduced different feature fusions into the SVM and RF detection models to achieve pixel-level detection of medical brain cancer hyperspectral data images. The main contributions of this study are as follows:

(1) This is the first time that the classification performance of machine models has been enhanced through a three - feature fusion method.
(2) The algorithm framework only requires 5 - pixel training, which solves the problem of the scarcity of medical datasets.
(3) Compared with neural networks, the algorithm framework maintains high precision while having lower training costs.

## Methods

### Dataset

#### Ethics statement.

This study was conducted using a publicly available hyperspectral medical imaging dataset. All data were fully anonymized prior to release, and no personally identifiable information was included. The use of the dataset complied with the terms and conditions specified by the data provider. Therefore, informed consent and additional ethical approval were not required.

#### Patient tumor characteristics and image.

The experiments were conducted using data gathered between March 2015 and June 2016 from the European project "HELICoiD" (Hyperspectral Imaging for Cancer Detection) at the University of Las Palmas de Gran Canaria in Spain. The dataset [14] comprised in vivo Multispectral Hyperspectral Imaging System (MHSIS) images of human brains, obtained during neurosurgical operations at the University Hospital of Gran Canaria. Patients, aged 18 and above, diagnosed with either primary or secondary brain tumors, including those with Grade IV Glioblastoma Multiforme (GBM), were involved in this study.During the surgery, a craniotomy was performed to expose the brain’s surface, followed by the acquisition of HS images using a hyperspectral imaging system. Based on the guidance from the Image-Guided Surgery (IGS) system, rubber ring markers were strategically placed to delineate areas of tumor and normal tissue.

The datasets, labeled 8-01, 12-01, 12-02, and 20-01, consisted of four hyperspectral medical images from three GBM patients. The first three digits of each dataset identifier corresponded to the patient’s unique number, while the last two digits denoted the image sequence within that patient’s set. Leveraging the expertise of neurosurgeons, pixels within each MHSI image were categorized and labeled to produce a reference image, distinguishing four classes: Tumor Tissue (TT), Normal Tissue (NT), Blood Vessel (BV), and Background (BG). The Background class specifically referred to the non-biological, disinfected rubber rings used for tumor and healthy brain area identification. A detailed pixel count for each dataset was presented in Table 1, with color-coded labels for Normal Tissue in green, Tumor Tissue in red, Blood Vessel in blue, and Background in yellow.

**Table 1:** Pixel quantity information for the four datasets.

Dataset	8-01	12-01	12-02	20-01
Total pixels	5477	15753	24464	9635
Normal tissue	2295	4516	6553	1842
Tumor tissue	1221	855	3139	3655
Blood vessel	1331	8697	6041	1513
Background	630	1685	8731	2625

For the model training, a random sampling method was employed, selecting five pixels per class to constitute the training set, thus streamlining the sampling process and ensuring a representative balance within the training samples. The remaining pixels were allocated to the test set for the evaluation of the trained model’s performance [15].

#### Image preprocessing.

**Fig 1:**
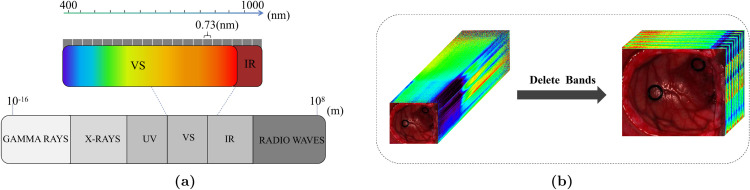
Data preprocessing flowchart. (a) Medical Dataset’s Spectral Information (b) Removal of noisy spectral bands.

The experiments utilized a Hyperspec VNIR series camera (HeadWall Photonics Inc., Fitchburg, MA, USA) for image generation. This camera is capable of capturing images within the spectral range of 400 to 1000 nm, with a sampling interval of 0.73 nm, as shown in Fig 1. The Hyperspectral Imaging system captures 826 spectral channels, covering the visible and near-infrared (VNIR) regions, with a spectral resolution of 2-3 nm. However, due to the lower performance of the HS sensor on certain channels, we excluded the extreme spectral channels, namely the first 56 and the last 126 channels, resulting in the use of 645 remaining spectral channels, as shown in Fig 1.

Lighting conditions in the natural environment are constantly changing [16], and hyperspectral acquisition sensors respond differently to light, leading to significant environmental variations in the collected hyperspectral data [17]. To avoid the spectral non-uniformity of illumination devices and the issue of dark current generated by camera sensors, as well as to eliminate the impact of external lighting conditions, we use Eq 1 to calibrate the image reflectance on a per-pixel basis, where ${img}_{𝑤}$ represents the white reference image obtained from a standard whiteboard, and ${img}_{𝑑}$ represents the dark reference image obtained with the camera shutter closed. By preprocessing to address the spectral non-uniformity of the illumination device and the dark current generated by the camera sensor, we remove noise from the spectral features and reduce the number of bands in the samples without losing relevant spectral information [18].

$${img}_{cal}=100\times \frac{img-{img}_{d}}{{img}_{w}-{img}_{d}}$$ (1)

### Feature extraction and fusion

#### Band selection.

Even after removing the interfering bands at the beginning and end of the spectral bands in hyperspectral images, due to the high correlation between bands in hyperspectral images, the processed medical hyperspectral data still suffers from data redundancy [19,20]. We decided to use a band selection algorithm to select more representative bands, retaining the useful information carried by the hyperspectral data, thereby reducing computational costs and time. Here, we chose the Data Gravitation and Weak Correlation-based Ranking algorithm [21] for band dimension reduction.

**Fig 2:**
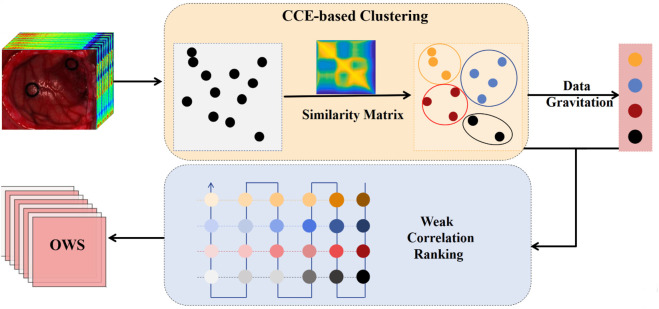
DGWCR flowchart.

The DGWCR algorithm, compared to traditional algorithms, can compress the dimension without destroying the original data structure and does not require manual annotation of samples. As shown in Fig 2, the Connection Centre Evolution (CCE) [22] algorithm is used to determine the clustering centers, and then, based on the data gravitation and the similarity matrix containing entropy, the most representative bands are selected within each cluster according to the data gravitation. Within each cluster, other bands are ranked according to weak correlation. Finally, a global ranking of the bands is obtained based on the S-shaped ranking strategy, and the top 20 bands are selected as the data for band selection.

#### Spectral indices.

After brain tissue becomes infected with cancer, the physiological characteristics of the tissue, such as proteins, lipids, and water content, [10,23,24] undergo changes, which in turn alter the reflectance of the corresponding location in the hyperspectral image. Spectral indices are tools used to describe certain spectral features, and by combining the reflectance of several specific wavelengths, they help analyze and understand the manifestation of different material components. To enhance the differences between tumor tissue and normal tissue, we selected 38 spectral indices related to tissue color, tissue structure, tissue physiology, and tissue water content from relevant literature, as shown in Table 2. We then used the Relief algorithm to identify spectral indices that can significantly differentiate tumors from these 38 indices.

**Table 2:** The names and formulas of 38 spectral indices.

No.	Spectral Indexs	Acronym	Equation
1	Pigments specific simple ratio	PSSRa	$R_{800}/R_{680}$
2	Pigments specific simple ratio	PSSRb	$R_{800}/R_{635}$
3	Red-green index	RGI	$R_{690}/R_{550}$
4	Ratio analysis of reflection of spectral chlorophyll a	RARSa	$R_{675}/R_{700}$
5	Ratio analysis of reflection of spectral chlorophyll b	RARSb	$R_{675}/(R_{700}\times R_{650})$
6	Ratio analysis of reflection of spectral chlorophyll c	RARSc	$R_{760}/R_{500}$
7	Photochemical reflectance index	PRI	$\left(R_{531}-R_{570}\right)/\left(R_{531}+R_{570}\right)$
8	Structure insensitive vegetation index	SIPI	$\left(R_{800}-R_{445})/(R_{800}+R_{680}\right)$
9	Normalized pigment chlorophyll index	NPCI	$\left(R_{680}-R_{430})/(R_{680}+R_{430}\right)$
10	Nitrogen reflectance index	NRI	$\left(R_{570}-R_{670})/(R_{570}+R_{670}\right)$
11	Normalized chlorophyll pigment ratio index	NCPI	$\left(R_{670}-R_{450})/(R_{670}+R_{450}\right)$
12	Plant pigment ratio	PPR	$\left(R_{550}-R_{450})/(R_{550}+R_{450}\right)$
13	Optimized soil-adjusted vegetation index	OSAVI	$(1+0.16)\times \left(R_{800}-R_{670}\right)/\left(R_{800}+R_{670}+0.16\right)$
14	Modified chlorophyll absorption ratio index	MCARI2	$\left((R_{750}-R_{705})-0.2\times (R_{750}-R_{550}))\times (R_{750}/R_{705}\right)$
15	Anthocyanin (Gitelson)	AntGitelson	$(1/R_{550}-1/R_{700})\times R_{780}$
16	Plant senescence reflectance index	PSRI	$(R_{660}-R_{510})/R_{760}$
17	Anthocyanin reflectance index	ARI	$1/R_{550}-1/R_{700}$
18	Structure Simple ratio	SR	$R_{900}/R_{680}$
19	Greenness index	GI	$R_{554}/R_{667}$
20	Narrow-band normalized difference	NBNDVI	$\left(R_{850}-R_{680})/(R_{850}+R_{680}\right)$
21	Normalized difference vegetation index	NDVI	$\left(R_{800}-R_{670})/(R_{800}+R_{670}\right)$
22	Red-edge NDVI	RNDVI	$\left(R_{750}-R_{705})/(R_{750}+R_{705}\right)$
23	Ratio vegetation structure index	RVSI	$(R_{712}-R752)/2-R_{732}$
24	Modified triangular vegetation index	MTVI	$12\times (1.2\times (R_{800}-R_{550})-2.5\times (R_{670}-R_{550}))$
25	Green NDVI	GNDVI	$\left(R_{750}-R_{540}+R_{570})/(R_{750}+R_{540}-R_{570}\right)$
26	Modified simple ratio	MSR	$\left(R_{800}/R_{670}-1)/(R_{800}/R_{670}+1\right)$
27	Triangular vegetation index	TVI	$0.5\times (120\times (R_{750}-R_{550})-200\times (R_{670}-R_{550}))$
28	Fluorescence ratio index 1	FRI1	$R_{690}/R_{630}$
29	Fluorescence ratio index 2	FRI2	$R_{750}/R_{800}$
30	Fluorescence ratio index 3	FRI3	$R_{690}/R_{600}$
31	Fluorescence ratio index 4	FRI4	$R_{740}/R_{800}$
32	Physiological reflectance index	PhRI	$\left(R_{550}-R_{531})/(R_{550}+R_{531}\right)$
33	Modified red-edge simple ratio index	mRESR	$\left(R_{750}-R_{445})/(R_{705}+R_{445}\right)$
34	Red-edge vegetation stress index 1	RVS1	$(R_{651}-R_{750})/2-R_{733}$
35	Red-edge vegetation stress index 2	RVS2	$(R_{651}-R_{750})/2-R_{751}$
36	Fluorescence curvature index	FCI	$R_{683}^{2}/(R_{675}\times R_{691}$)
37	Red edge position	RRE	$(R_{670}-R_{780})/2$
38	Water band index	WBI	$R_{900}/R_{970}$

Relief algorithm is a feature weighting algorithm that assigns different weights to features based on their correlation with the class labels, and features with weights below a certain threshold are removed [25–27]. The Relief algorithm randomly selects a sample *R* from the training sample set each time, and then identifies *k* nearest neighbor samples (near Hits) from the same class as *R*, and *k* nearest neighbor samples (near Misses) from each different class of *R*. It then updates the weights of each feature according to Eq 2. Here, diff(A,${𝑅}_{1}$, ${𝑅}_{2}$) represents the difference in feature A between samples ${𝑅}_{1}$ and ${𝑅}_{2}$ (Eq 3). ${𝑅}_{𝑖}$ is the sample from the i-th sampling, ${𝐻}_{𝑗}$ is the j-th nearest neighbor of ${𝑅}_{𝑖}$, *m* is the number of samplings, *k* is the number of nearest neighbors, P(C) is the probability of the occurrence of class *C*, Class(R) is the class to which the random sample *R* belongs, P(Class(R)) is the probability of the occurrence of the random sample *R*, and *M*_*j*_(*C*) indicates the j-th nearest neighbor sample that does not belong to Class(*R*) in class *C*.

$$W(I)=W(I)-\sum_{C=\mathrm{Class}(R)}\sum_{j=1}^{k}\frac{\mathrm{diff}\;\left(I,R_{i},H_{j}\right)}{mk}\;\;+\sum_{C\neq \mathrm{Class}(R)}\left[\frac{p(C)}{1-p\;\left(\mathrm{Class}(R_{i})\right)}\sum_{j=1}^{k}\mathrm{diff}\;\left(I,R_{i},M_{j}(C)\right)\right]/(mk)$$ (2)

$$\mathrm{diff}(A,R_{1},R_{2})=\left\{ \begin{array}{cc} \frac{\left|R_{1}[A]-R_{2}[A]\right|}{\max(A)-\min(A)}, & \text{if}Aiscontinuous\}\\ 0, & \text{if}Aisdiscreteand\}R_{1}[A]=R_{2}[A]\\ 1, & \text{if}Aisdiscreteand\}R_{1}[A]\neq R_{2}[A] \end{array}\right.$$ (3)

**Fig 3:**
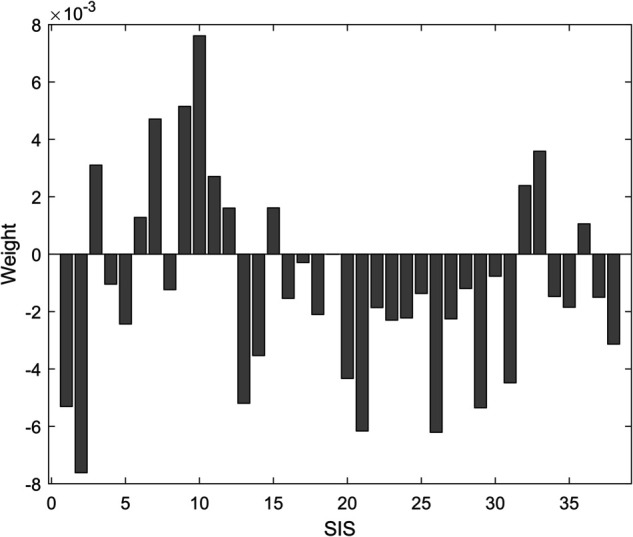
Weight map of 38 spectral indices obtained from the relief algorithm.

We selected 70 hyperspectral images of tumors, of which 38 images contained tumors and 32 were images of normal tissue. We labeled whether they contained tumors with 0 and 1, which will serve as training labels. By averaging the reflectance of each band, we obtained the average reflectance spectrum of the hyperspectral image, and then calculated its 38 spectral indices from the spectral curve. By calculating the correlation between these 38 spectral indices and the presence of tumors, we obtained a weight diagram as shown in Fig 3. We selected the top 5 spectral indices most sensitive to tumors, RGI, PRI, NPCI, NRI, and mRESR, as the spectral index data.

#### Texture features.

Hyperspectral images contain not only rich spectral information but also important spatial information. Textural features can capture the spatial information in brain cancer, including the shape, size, and distribution of the cancer, which is crucial for distinguishing different tissues with similar spectral characteristics [28]. In hyperspectral imaging, due to the lower spatial resolution and a higher number of mixed pixels, detection relying solely on spectral information may not be accurate enough. Moreover, in some cases, different tissues may have similar spectral features, or the spectral characteristics of the same tissue may vary significantly under different conditions. Textural features provide an additional dimension of information that helps address these issues, leading to more accurate tumor identification. Furthermore, textural features are somewhat adaptable to changes in image acquisition conditions, such as lighting and shadows, making them useful for detection information under variable environmental conditions. Therefore, we use the Invariant Attribute Profiles (IAPS) algorithm to extract textural features, enhancing the model’s ability to recognize brain cancer tissue from a spatial dimension.

The IAPS algorithm processes spatial information by applying isotropic filters or convolution kernels to hyperspectral images to extract features that are invariant to translation and rotation. It then employs the Simple Linear Iterative Clustering (SLIC) superpixel segmentation [29,30] technique to divide the image into regions with similar characteristics, forming clusters. In the spectral frequency domain, features from the spatial domain are subjected to a Fourier transform, converting the image from the spatial domain to the frequency domain, and simultaneously transforming the Fourier-transformed image from the Cartesian coordinate system to the polar coordinate system. Under polar coordinates, by performing a polar Fourier transform on each pixel of the input image, we can obtain m amplitude features corresponding to *m* different Fourier orders with each pixel (Eq 4).

$${ℱ}_{m}^{\left(1\right)}(x,y)=\left\|D_{m}(x,y)\right\|,\;m=0,1,\ldots ,M$$ (4)

Eq 5) obtain absolute rotation-invariant features by removing the phase information.

$${ℱ}_{m}^{2}(x,y)=F_{m}(x,y)*F_{m^{\prime }(x,y)}$$ (5)

To mitigate the loss of rich phase information, relative rotation-invariant features are developed by coupling the obtained additional convolutional Fourier representation with two adjacent convolution kernel radii (Eq 6).

$${ℱ}_{m}^{3}(x,y)\;=\frac{\left(F_{m}(x,y)*F_{m^{\prime },r1}(x,y)\right)\overset{―}{\left(F_{m}(x,y)*F_{m^{\prime },r2}(x,y)\right)}}{\sqrt{\|\left(F_{m}(x,y)*F_{m^{\prime },r1}(x,y)\right)\overset{―}{\left(F_{m}(x,y)*F_{m^{\prime },r2}(x,y)\right)\|}}}$$ (6)

We integrate the three components as shown in Eq 7.

$${ℱ}_{PWFF}(x,y)=[{ℱ}_{0}^{1}(x,y),\ldots ,{ℱ}_{m}^{1}(x,y),\ldots ,{ℱ}_{0}^{2}(x,y)\;\;\ldots ,{ℱ}_{m}^{2}(x,y),\ldots ,{ℱ}_{0}^{3}(x,y),\ldots ,{ℱ}_{m}^{3}(x,y)]$$ (7)

Using isotropic triangular convolution kernels to aggregate the Polarimetric Weighted Fractal Features (PWFF) to obtain frequency-domain invariant features, and finally stacking the features extracted from the spatial and frequency domains to form the final Invariant Attribute Profiles (IAPs) feature representation. Through the acquisition of IAPs, a robust texture feature extraction can be provided for the model. In terms of textural features, the top 5 dimensions ranked by IAPS are selected as the textural feature data.

#### Feature fusion.

Feature fusion is a pivotal concept in the realms of machine learning and data analysis, with significant applications in image processing, computer vision, and medical image analysis. This process entails the amalgamation of features derived from various sources or stages of processing. The objective of feature fusion is to bolster the model’s capacity to generalize by harnessing the strengths of diverse features, thereby enhancing the model’s comprehension of the data and elevating the precision of classification, detection, or predictive tasks. We have implemented feature fusion in hyperspectral tumor detection, and to substantiate the notion that the integration of three distinct data modalities can augment the precision of medical hyperspectral detection, we conducted a comparative analysis of detection accuracy across seven feature configurations: three individual features (OWS,SIS,TFS), three combined pairs of these features (OWS+SIS, OWS+TFS, SIS+TFS), and the comprehensive fusion of all three features (ALL). The method of combination employed here involves a concatenation along the third dimension.

### Classifier and evaluation

#### Classifier selection.

Currently popular medical hyperspectral detection models often come with built-in model selection and texture acquisition capabilities. To avoid the impact of complex algorithms on the fusion of the three types of data, we choose SVM (Support Vector Machine) and RF (Random Forest), two common and simple algorithms, to prevent the hyperspectral detection from gaining an advantage from the model itself. In hyperspectral image detection, SVM can effectively identify the spectral characteristics of different materials and achieve high-precision detection by selecting the appropriate kernel function and parameters. RF, as an ensemble learning method, has demonstrated its powerful capabilities in past medical hyperspectral image detections. It constructs multiple decision trees and aggregates their predictions, effectively handling high-dimensional data and capturing complex patterns within the data. In the experiment, cross-validation methods were used to find the optimal penalty parameter C and kernel function parameter gamma for SVM, and a random forest composed of 200 decision trees was used. This experiment was debugged in MATLAB 2020a and run on a personal computer with an Intel Core i7-12650H central processor and 16GB of RAM.

#### Evaluation metrics.

In this study, we employed three widely recognized standards to assess the performance of pixel-level detection in medical images: Overall Accuracy, Average Accuracy, and the Kappa coefficient [15]. These evaluation metrics provide us with an intuitive method for quantifying the results of our experiments and are frequently used in research within the relevant field. Overall Accuracy is a metric that quickly gives an overview of the model’s average performance across all categories. It measures the proportion of correctly detected samples out of the total samples, reflecting the detector’s overall performance on all categories. Average Accuracy takes into account the detection effect of each category, calculating the average of the accuracies of each category. AA is particularly important for dealing with imbalanced datasets because it can reveal the model’s performance differences across various categories, helping us identify the strengths and weaknesses of the model. The Kappa coefficient is a statistical measure used to assess the performance of a detector while considering the possibility of random detection. The value of the Kappa coefficient ranges from –1 to 1, with 1 indicating perfect agreement and 0 indicating that the detector’s performance is no different from random detection. The advantage of this coefficient is that it accounts for random agreement, thus providing an accurate assessment of the detector’s performance even in cases where the category distribution is uneven. By considering these three metrics comprehensively, we can evaluate the performance of medical image detection models in a holistic manner, ensuring that our research results are both scientifically sound and practically valuable.

### Workflow of the study

**Fig 4:**
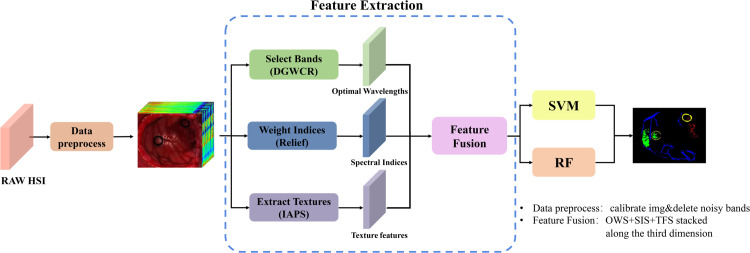
Main flowchart of the hyperspectral tumor image classification process.

In this study, we developed a machine learning algorithm for hyperspectral image (HSI) classification by fusing multiple types of features (Fig 4). The fusion integrates information from three complementary aspects: wavelength selection, disease indices, and texture features. Using this approach, the algorithm accurately delineates tumor locations and boundaries across different glioma HSIs. Moreover, the effectiveness and generalizability of the three-feature fusion in enhancing model accuracy were validated using two distinct machine learning models.

## Results

### Performance of the model on SVM

Our algorithm was implemented with SVM classifier across four datasets, conducting experiments to validate the efficacy of the three-feature fusion approach and comparing it with other feature combinations. To provide a more intuitive understanding of the classification effects under different feature inputs, we visualized the classification results for seven types of inputs. For the sake of brevity in the manuscript, only the classification effect diagrams for different inputs of dataset 8-01 were displayed.

**Fig 5:**
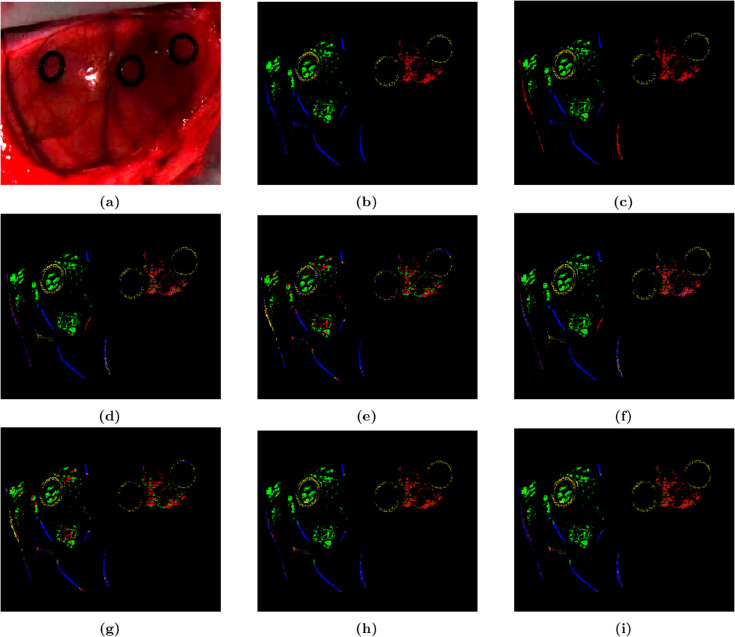
Prediction effect diagrams of SVM with 8-01 dataset based on different feature inputs. (a) False color image. (b) Ground truth image. (c) OWS. (d) SIS. (e) TFS. (f) OWS + SIS. (g) OWS + TFS. (h) SIS + TFS. (i) All features combined.

As shown in Table 3, for the dataset 8-01, the three-feature fusion input achieved the best performance with OA, AA, and KA of 91.86%, 91.68%, and 0.8941, respectively. Among the single features, OWS achieved the best overall accuracy with 89.48%, and the three-feature fusion improved this by 2.4%. In the two-feature fusions, the three-feature fusion led with an increase of 0.3% over TFS+SIS, 5.79% over OWS+SIS, and 19.53% over OWS+TFS. The three-feature fusion also showed varying levels of improvement in AA and KA. As illustrated in Fig 5, Fig 5i, which represented the detection map for SVM with three-feature fusion input, more closely matched the ground truth compared to Fig 5b–5h. Only a small number of vascular pixels were misclassified as other categories in Fig 5i. The judgment on the tumor was completely consistent with Fig 5b.

**Table 3:** Detection accuracies of OA, AA, and KA for SVM with different feature inputs on four datasets.

Dataset	Evaluation	OWS	SIS	TFS	OWS+SIS	OWS+TFS	TFS+SIS	ALL
8-01	OA	0.8948	0.8602	0.6976	0.8607	0.7233	0.9175	**0.9186**
	AA	0.8929	0.8529	0.6885	0.8536	0.7117	0.9155	**0.9168**
	KA	0.8637	0.8175	0.6043	0.8182	0.6381	0.8927	**0.8941**
12-01	OA	0.8486	0.8394	0.5554	0.8400	0.8098	0.9447	**0.9469**
	AA	0.9203	0.8931	0.6413	0.8956	0.8284	0.9432	**0.9453**
	KA	0.8065	0.7913	0.4106	0.7918	0.7554	0.9300	**0.9327**
12-02	OA	0.8381	0.9165	0.5763	0.9181	0.6202	0.9510	**0.9557**
	AA	0.8589	0.9255	0.5957	0.9267	0.6558	0.9525	**0.9562**
	KA	0.7861	0.8897	0.4359	0.8918	0.4941	0.9354	**0.9415**
20-01	OA	0.9393	0.9279	0.6318	0.8936	0.8226	0.9538	**0.9595**
	AA	0.9075	0.8976	0.6382	0.8625	0.7977	0.9357	**0.9385**
	KA	0.9204	0.9057	0.5111	0.8615	0.7672	0.9394	**0.9469**

**Fig 6:**
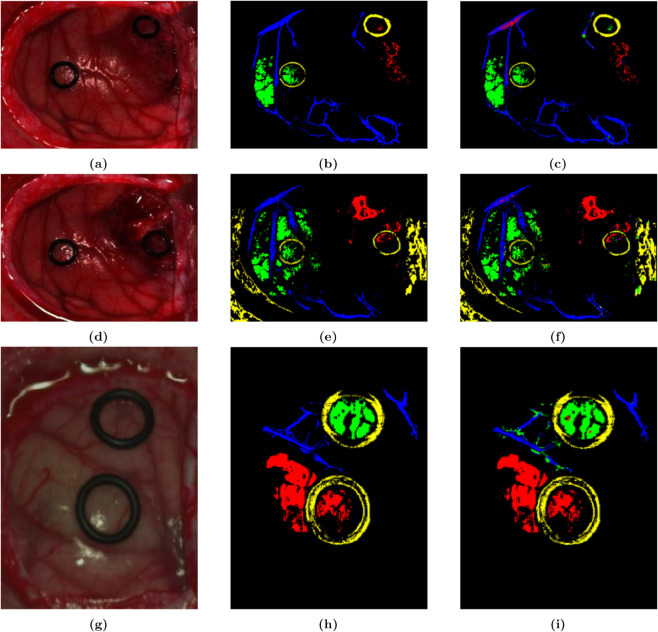
Color images of living human brain tissue from three patients, their corresponding grayscale images, and SVM detection effect diagrams with three-feature fusion. (a, b, c) Images from patient 12-01. (d, e, f) Images from patient 12-02. (g, h, i) Images from patient 20-01.

The SVM detector achieved the best performance in terms of OA, AA, and KA with scores of 96.49%, 94.53%, and 0.9327 respectively, when using three feature fusion inputs in the 12-01 dataset. In the 12-02 dataset, the three-feature fusion reached the maximum values of OA, AA, and KA with 95.57%, 95.62%, and 0.9415 respectively. In the 20-01 dataset, the three-feature fusion obtained the maximum values of OA, AA, and KA with 95.95%, 93.85%, and 0.9469 respectively. The consistent performance across various datasets demonstrated the reliability of the three-feature fusion in enhancing tumor detection accuracy. As shown in Fig 6, the detection effect diagram with three-feature input showed minimal error when compared to the ground truth. In terms of tumor pixel detection, aside from some errors within the yellow circle in the upper right corner of Fig 6c, the results were consistent with the corresponding actual ground truth images.

### Performance of the model on RF

To verify the detection accuracy and effectiveness on RF, we also conducted experiments on four Multi-Hyppectral Imaging (MHSI) datasets, using OA, AA, and KA as metrics for accuracy assessment, as shown in Table 4. Similarly, we only visualized the detection effect diagrams of the seven types of inputs on one dataset for comparison.

**Table 4:** Detection accuracies of OA, AA, and KA for RF with different feature inputs on four datasets.

Dataset	Evaluation	OWS	SIS	TFS	OWS+SIS	OWS+TFS	TFS+SIS	ALL
8-01	OA	0.9732	0.8039	0.6795	0.9786	0.9901	0.8937	**0.9921**
	AA	0.9740	0.7654	0.6537	0.9789	0.9918	0.8533	**0.9928**
	KA	0.9650	0.7483	0.5792	0.9720	0.9871	0.8632	**0.9897**
12-01	OA	0.8668	0.8870	0.5864	0.8945	0.8687	0.8589	**0.9095**
	AA	**0.9103**	0.8417	0.6217	0.8945	0.8746	0.8194	0.8950
	KA	0.8280	0.8567	0.4538	0.8654	0.8317	0.8200	**0.8852**
12-02	OA	0.7991	0.8876	0.4126	0.8809	0.8145	0.8720	**0.8956**
	AA	0.8250	0.8941	0.4317	0.9026	0.8424	0.8831	**0.9134**
	KA	0.7348	0.8517	0.2183	0.8421	0.7544	0.8313	**0.8616**
20-01	OA	0.9174	0.8551	0.6180	0.9289	0.9221	0.8903	**0.9335**
	AA	0.8846	0.8205	0.6069	0.9002	0.8921	0.8641	**0.9052**
	KA	0.8920	0.8114	0.4939	0.9070	0.8981	0.8569	**0.9131**

**Fig 7:**
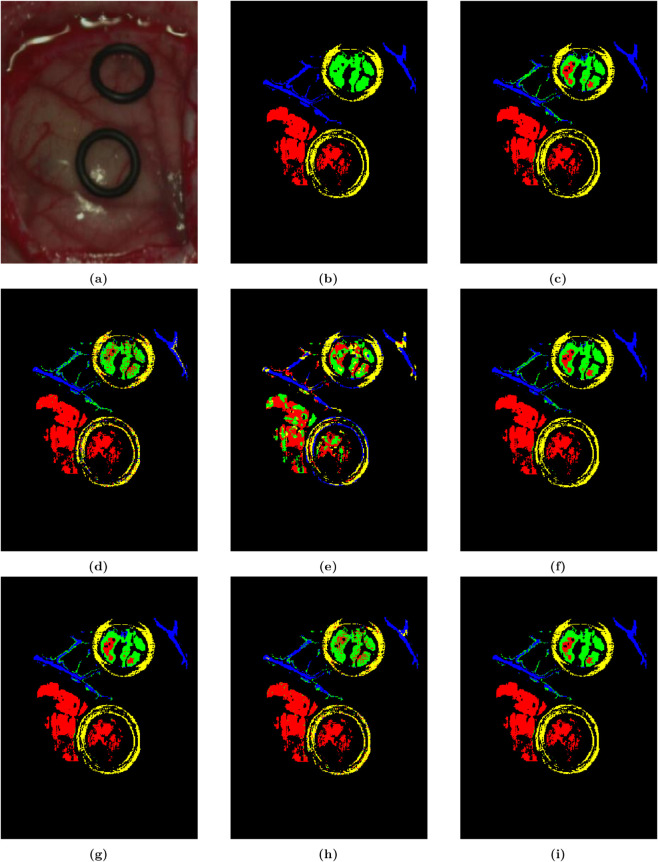
Prediction effect diagrams of RF with 20-01 dataset based on different feature inputs. (a) False color image, (b) Ground truth image, (c) OWS, (d) SIS, (e) TFS, (f) OWS + SIS, (g) OWS + TFS, (h) SIS + TFS, (i) All features combined.

In the RF detector, the 8-01 dataset achieved the highest metrics in OA, AA, and KA with 99.21%, 99.28%, and 0.9897, respectively. For the 12-01 dataset, RF achieved a detection accuracy higher than other inputs with 90.95% OA, 0.8852 KA. For the 12-02 dataset, RF also outperformed other inputs with the same metrics of 89.56% OA, 91.34% AA, and 0.8616 KA. The detection effect diagrams for the three datasets were shown in Fig 7. On the 12-01 dataset, under the yellow rubber band in the upper right corner, only some tumor pixels were incorrectly detected as other tissues. On the 8-01 and 12-02 datasets, tumor pixels were perfectly detected by the model.

**Fig 8:**
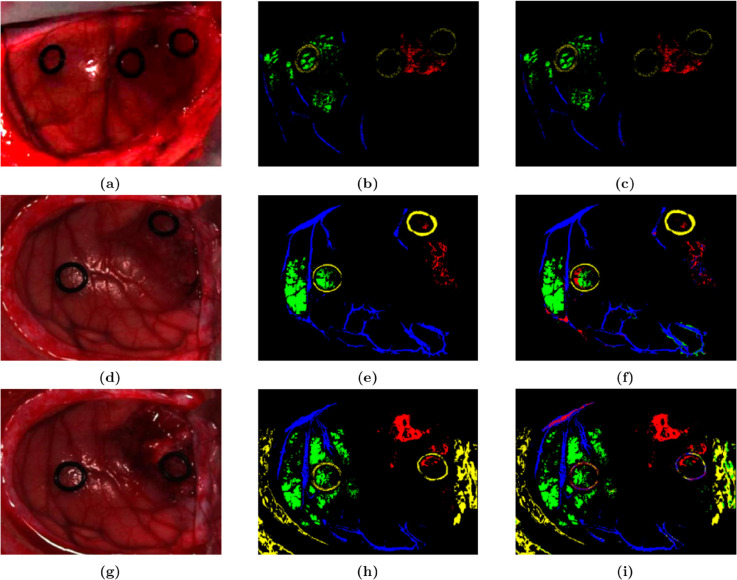
Color images of living human brain tissue from three patients, their corresponding grayscale images, and RF detection effect diagrams with three-feature fusion. (a, b, c) Images from the 8-01 dataset. (d, e, f) Images from the 12-01 dataset. (g, h, i) Images from the 12-02 dataset.

The detection accuracy of the 20-01 dataset on the RF was shown in Table 4. The RF led other inputs by an average of about 8% in the OA metric with 93.35%, by about 7% in the AA with 90.52%, and by about 0.1 in the KA with 0.9131. We trained the corresponding models for the seven types of inputs. Fig 8i showed the predicted detection diagram of the RF with three-feature fusion; it was consistent with Fig 8b except for some errors in the upper right rubber band and the middle blood vessel. Compared to the detection performance (Fig 8c–8h) under the other six inputs, the detection effect diagram with three-feature fusion showed superior performance in identifying the location of the tumor for Fig 8a.

### Summary of model classification performance

**Fig 9:**
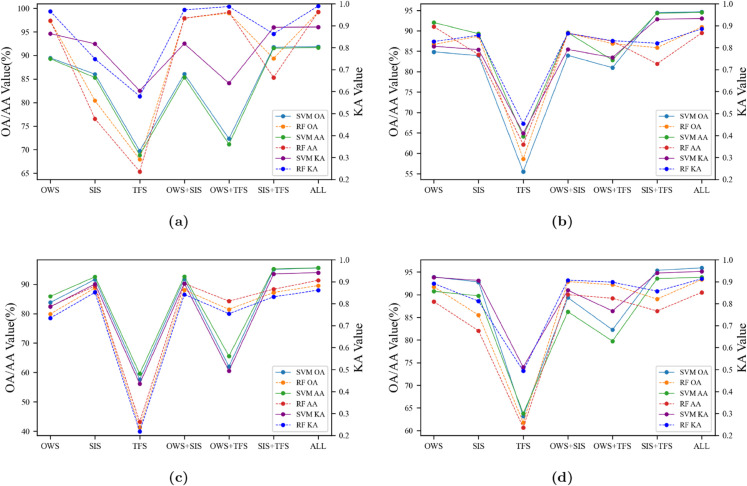
SVM and RF detection accuracy on different datasets: (a) 8-01, (b) 12-01, (c) 12-02, (d) 20-01.

To validate the performance of a hyperspectral tumor image detection algorithm based on the fusion of multiple features, we conducted comparisons of seven different feature combinations on four medical hyperspectral datasets using two detectors. The evaluation was performed using three assessment metrics, and the statistical effects were shown in Fig 9.

Regarding the seven data combinations, it could be observed that the three basic single data inputs, particularly the optimal wavelength parts, yield the best detection effects, which was entirely reasonable. Spectral indices and textural features primarily served an auxiliary role; spectral indices aimed to enhance the data’s sensitivity to tumor tissues, while textural features provided additional spatial information. From Fig 9, it could be observed that the overall trend for the four datasets showed that the fusion of features performed better than a single input, with the three-feature fusion showing the most outstanding performance.

In terms of detectors, the performance of SVM and RF was not consistent across different datasets; sometimes RF outperforms, and other times SVM was better. We believed this was due to minor differences in the datasets. The purpose of using two detectors was to avoid the improvement in accuracy being specific to a particular detector; in practical applications, only one detector could be used.

## Discussion

In this study, we are committed to developing a hyperspectral tumor image detection framework suitable for clinical use, to meet the need for precise identification of brain tumor locations. This framework significantly improves the accuracy of tumor identification by integrating three key data types: wavelength selection, spectral indices, and textural features, thereby achieving precise localization of tumors.

Hyperspectral imaging technology, with its outstanding capabilities, is increasingly valued in the medical field [9,11]. It not only extracts the spectral characteristics of each pixel but also provides detailed information about the different tissue components and their spatial distribution. At specific wavelengths, tissues with different pathological states exhibit different reflectance, absorption, and electromagnetic energy characteristics due to differences in chemical composition and physical properties, which are manifested through characteristic spectra. By carefully analyzing these spectral signals, we can achieve qualitative or quantitative detection of tissue conditions. Combined with the spatial distribution information provided by hyperspectral images, we can visualize various pathological states of tissues and diagnose the disease conditions of tissues. In recent years, the application of hyperspectral technology in the field of medical diagnosis has gradually increased [31]. For example, Song Nan and others successfully identified the spectral differences between gliomas and normal brain tissues by deeply analyzing and processing hyperspectral data, providing a new method for distinguishing between gliomas and normal brain tissues. Yi Weisong and others used a near-infrared hyperspectral imaging system to obtain hyperspectral images of gastric cancer tissues and normal tissues [32], extracted the characteristic spectra of tumors and normal tissues by chemometric methods, and performed spectral attribution. They further combined multiple target recognition algorithms to automatically extract gastric cancer tumor targets, and ultimately used image fusion technology to visually and intuitively display the recognition results, forming a method system for diagnosing gastric cancer tissues with hyperspectral images. These studies show that hyperspectral imaging technology is not only highly credible in tumor identification but also can provide more accurate and in-depth diagnostic information [33].

In the field of hyperspectral image detection, the application of deep learning has been very extensive [34–36], and many researchers have proposed many innovative and powerful frameworks, which have been effectively verified on remote sensing datasets [37–39]. For example, Li Ming-song fused the central pixel and neighboring pixels through the network to achieve detection [40], while Zheng Zhuo designed a lateral connection module to connect the encoder and decoder, thereby fusing the spatial details in the encoder and the semantic features in the decoder, [41] etc. In the field of hyperspectral medical image detection, although many innovations focus on the optimization of deep learning network architecture, such as introducing residual connections, attention mechanisms, encoding and decoding, and other advanced technologies to improve detection accuracy, our research focus has shifted to innovation at the data level. We are committed to exploring how to enhance the detection capabilities of the model through diversified processing and feature fusion of raw hyperspectral data [42,43]. This innovative method, which starts from the data, aims to provide new solutions for the precise detection of hyperspectral medical images by deepening data understanding and feature extraction. We try to process raw hyperspectral data in various ways and improve detection accuracy through feature fusion. Feature fusion is a machine learning technique that merges features from different sources to form a better feature representation, thereby enhancing model performance [44]. In the spectral analysis of medical images, optimal wavelength features correspond to the spectral absorption or reflection characteristics of tumor tissues [45], which can provide highly specific identification of the lesion area. Spectral indices can help enhancing the model’s sensitivity to tumor tissues [46], especially in distinguishing between tumors and normal tissues. Textural features reflect the spatial heterogeneity of tissue structure, providing important morphological information for the image [47], which helps to identify the boundaries and morphological characteristics of tumors. By combining the advantages of the three features, enhancing the model’s understanding of the data, and thus improving the model’s accuracy.

To select the appropriate bands for feature fusion, we used the DGWCR algorithm [21] to screen 20 representative bands from a large number of bands, forming the data basis for optimal wavelength. To enhance the model’s sensitivity to tumor tissue, we used the Relief algorithm [48] to select 5 spectral indices most related to tumor tissue from 38 spectral indices, as the data part of the spectral index. At the same time, to provide rich spatial texture information, we applied the IAPS algorithm [49] to calculate 5-dimensional regional texture features. By using band selection as the basis, texture features provide spatial auxiliary information, and spectral index data enhance the sensitivity to tumor tissue, we try to improve the performance of the detection model by splicing the three types of data. This method not only innovates the way of data processing but also is expected to achieve higher precision diagnosis in the field of hyperspectral medical image detection, bringing new perspectives and possibilities for clinical applications.

To verify the effect of the three-feature fusion on improving detection accuracy, we used two widely used detectors in brain cancer detection and analyzed them separately using three independent features and their fused features. In the early stage of the experiment, we conducted a detailed preprocessing of hyperspectral data to eliminate the impact of environmental and equipment factors. Based on the selected optimal wavelengths, spectral indices, and textural features, we constructed SVM and RF detection models [50,51] and detected tumor hyperspectral images using seven different input combinations and two detectors. We used standard evaluation indicators, including overall accuracy, average accuracy, and Kappa coefficient, to comprehensively evaluate the detection results. In addition, to intuitively display the detection effect, we visualized each detection result. The experimental results show that on four different datasets and two detectors, the detection method that fuses three features is superior to the other six single or two-way fusion input methods in all detection indicators, achieving excellent detection results. In the experimental design, we deliberately chose a variety of datasets to avoid the accidental results that a single dataset might bring; at the same time, we used two different detectors to exclude the impact of the detector’s own characteristics on the improvement of accuracy. These experimental designs ensure the reliability and universality of our results. Ultimately, the experimental results prove that under the combined action of optimal wavelengths, spectral indices, and textural features, the detection accuracy and performance of hyperspectral data have been significantly improved, and this comprehensive method provides a powerful auxiliary tool for doctors to identify tumor locations.

Although our research has achieved certain results, there are still some limitations. First, the datasets we used are public and only include patients with IV glioblastoma, which may lead to sample selection bias and limit the universal applicability of the research results. To overcome this limitation, we plan to include more data from patients with different types of tumors, different ages, and genders in future research to ensure the accuracy and wide applicability of the research. Secondly, on the algorithm level, although the DGWCR, Relief, and IAPS algorithms played an important role in this study, there is still room for further optimization. We consider replacing these algorithms with algorithms that can more efficiently extract effective data and speed up processing to improve the response speed and practicality of the entire framework. Overall, we are committed to building a powerful, universal, and easily promoted framework based on the fusion of optimal wavelengths, spectral indices, and textural features to achieve precise localization of tumor positions and assist doctors in tumor resection surgery. This study, as a high-quality study in the field of medical hyperspectral imaging and artificial intelligence, shows great clinical application potential. We believe that through continuous technological innovation and optimization, our research will bring new breakthroughs to the fields of medical image analysis and tumor diagnosis, providing patients with more accurate and personalized treatment plans.
